# Comparison of Chemical Composition and Antioxidant Capacity of Fruit, Flower and Bark of *Viburnum opulus*

**DOI:** 10.1007/s11130-019-00759-1

**Published:** 2019-07-19

**Authors:** Dominika Polka, Anna Podsędek, Maria Koziołkiewicz

**Affiliations:** Institute of Technical Biochemistry, Department of Biotechnology and Food Sciences, Lodz University of Technology, Stefanowskiego 4/10, 90-924 Łódź, Poland

**Keywords:** *Viburnum opulus*, Fruit, Flower, Bark, Macronutrients, Antioxidants

## Abstract

In this work, the profiles of phenolics, fiber, pectins, sugars, organic acids and carotenoids, vitamin C, ash, protein and fat contents, as well as antioxidant capacity were compared in fruits, flowers, and bark of *Viburnum opulus* (VO). Antioxidant capacity was evaluated against ABTS, hydroxyl, peroxyl and superoxide free radicals, and as a reducing power by using *in vitro* test. The results showed great quantitative differences in the composition of the VO morphological parts tested. Fruits contained the highest concentrations of fat, organic acids, sugars, soluble dietary fiber (10.57 ± 0.54; 7.34 ± 0.06; 32.27 ± 1.25; 6.82 ± 0.38 g/100 g DW, respectively) and carotenoids (2.70 ± 0.07 mg/100 g DW). Whereas, the bark exceeded the remaining parts of the VO in terms of antioxidant capacity, ash (9.32 ± 0.17 g/100 g DW), total (59.34 ± 0.75 g/100 g DW) and insoluble dietary fiber (58.20 ± 0.73 g/100 g DW) contents as well as phenolic compounds (3.98 ± 0.04 g/100 g DW). Among the phenolic compounds quantified in this study, chlorogenic acid and (+)-catechin had the highest concentrations (> 1 g/100 g DW) in the flowers and bark, respectively.

**Electronic supplementary material:**

The online version of this article (10.1007/s11130-019-00759-1) contains supplementary material, which is available to authorized users.

## Introduction

*Viburnum opulus* L. (Adoxaceae), commonly known as European guelder, is also called as European cranberrybush, guelder rose, wild guelder rose, cherry-wood, rose elder, crampbark tree and snowball bush, and gilaburu in Turkey [1–3]. It is widespread in Europe, North and Central Asia, and North Africa [2, 4]. *Viburnum opulus* (VO) is a valuable decorative, medicinal and food plant. In Russia, Ukraine and among many Siberian nations the VO fruits, despite their astringent-bitter-sour taste, are used in traditional cuisine as a component of marmalades, jams, cordials and liqueurs, and “Kalinnikov” pies as well as herbal teas [5]. Also, in Scandinavia the fruits are popular when cooked into preserves while in Canada they may replace cranberries [3]. In the Central Anatolia region of Turkey juice from VO fruits is produced as a commercial product [6].

The VO fruits and fruit juice have been used to treat a wide range of illnesses, including bleeding, heart disease, high blood pressure, coughs and cold, neurosis and diabetes [3, 7–9]. In animal studies, extracts of VO fruits prevented male reproductive system against damages caused by taxanes and showed anti-endometriotic activity [6, 10]. Moreover, *in vitro* studies demonstrated the antioxidant properties of VO fruit, branch, leaf and bark extracts [11–16]. Health benefits of *V. opulus* result from the presence of bioactive components in the plant, such as phenolic compounds, vitamin C, carotenoids, triterpenes, iridoids, essential oils, saponins and dietary fiber [5, 16–18]. As reported by several authors the levels of bioactive compounds vary between fruit genotypes and parts of plant [1, 18, 19].

So far, most of the research have been carried out to characterize the chemical composition of VO fruit and fruit juice. However, there is no information or little is known about the chemical characteristic of other parts of the plant for which health-promoting effects have also been demonstrated. The aim of this study is to compare the biochemical components and antioxidant potential of poorly described flowers and bark with well known fruits of VO to recommend the use of the most valuable VO parts in pharmaceutics, cosmetics and/or functional foods.

## Materials and Methods

The material and methods section is presented as supplementary material 1.

## Results and Discussion

### Comparison of Macronutrients of VO Fruits, Flowers and Bark

To our best knowledge, there are no reports on the basic chemical composition of VO bark and flowers. However, the content of macronutrients in VO fruits was previously analyzed by other authors [1, 4, 18–20]. The concentrations of the major components of the different morphological parts of VO are given in Table 1. The statistically significant differences (*p* < 0.05) between VO fruits, bark and flowers in terms of protein, ash, organic acids, sugars, dietary fiber and pectin contents were observed. The fruit of VO was characterized by the significantly highest content of fat (10.57 g/100 g DW), total organic acids (7.34 g/100 g DW) and total sugars (32.27 g/100 g DW) as well as the lowest concentration of ash (2.96 g/100 g DW) and total fiber (38.44 g/100 g DW). Meanwhile, the flower of VO was the richest in protein and pectins (9.72 and 8.58 g/100 g DW, respectively). Among three VO anatomical parts tested bark had the highest content of ash (9.32 g/100 g DW) and total fiber (59.34 g/100 g DW). In the fresh VO fruits grown in Turkey the ratio of protein to ash was 1.8 and it was consistent with the value calculated for the VO dried fruit tested in this work [20]. In addition, the VO fruits tested were comparable to other fruits in terms of ash and protein contents, but exceeded them in terms of fat and fiber contents [21]. Whereas, VO flowers were characterized by a similar content of protein to Roselle flowers [22]. On the other hand, the content of ash in VO flowers was 2–3 times lower than in the mentioned above flowers. Fereira et al. [23] obtained the larger amount of ash (14.6 g/100 g DW) in *Quercus faginea* bark in comparison to our result for VO bark.

**Table 1:** The elementary chemical composition of dried flowers, bark and fruits of *Viburnum opulus*

Factor	Flowers	Bark	Fruits
	g/100 g dried weight (DW)		
Ash	4.07 ± 0.06^b^	9.32 ± 0.17^c^	2.96 ± 0.22^a^
Protein	9.72 ± 0.53^c^	3.26 ± 0.10^a^	5.40 ± 0.16^b^
Fat	5.39 ± 0.26^a^	10.06 ± 0.01^b^	10.57 ± 0.54^b^
Organic acids total	1.81 ± 0.02^a^	1.84 ± 0.05^a^	7.34 ± 0.06^b^
Oxalic acid	0.54 ± 0,02^a^	0.85 ± 0.01^b^	–
Citric acid	–	–	3.09 ± 0.01
Tartaric acid	0.18 ± 0.01^a^	–	0.37 ± 0.02^b^
Malic acid	0.61 ± 0.03^a^	–	3.13 ± 0.02^b^
Quinic acid	–	–	0.75 ± 0.04
Succinic acid	0.48 ± 0.03^a^	0.97 ± 0.06^b^	–
Fumaric acid	–	0.02 ± 0.00	–
Sugars total	11.92 ± 0.41^b^	1.52 ± 0.10^a^	32.27 ± 1.25^c^
Fructose	4.57 ± 0.01^a^	–	10.72 ± 0.09^b^
Glucose	2.01 ± 0.06^a^	–	15.29 ± 0.74^b^
Sucrose	5.34 ± 0.35^b^	1.52 ± 0.10^a^	6.26 ± 0.43^c^
Fiber total	45.39 ± 2.07^b^	59.34 ± 0.75^c^	38.44 ± 0.41^a^
SDF UA	1.87 ± 0.12^b^	0.19 ± 0.01^a^	2.10 ± 0.08^c^
SDF NS	1.06 ± 0.05^a^	0.95 ± 0.05^a^	4.72 ± 0.30^b^
SDF total	2.93 ± 0.16^b^	1.13 ± 0.06^a^	6.82 ± 0.38^c^
IDF UA	0.35 ± 0.03^a^	9.93 ± 0.26^c^	2.35 ± 0.10^b^
IDF NS	12.61 ± 0.21^b^	22.60 ± 1.11^c^	9.72 ± 0.16^a^
IDF KL	29.50 ± 2.23^c^	25.67 ± 1.71^b^	19.54 ± 0.24^a^
IDF total	42.46 ± 2.05^b^	58.20 ± 0.73^c^	31.62 ± 0.18^a^
Pectin total	8.58 ± 0.29^c^	4.15 ± 0.06^a^	6.23 ± 0.26^b^
WSP	1,96 ± 0.15^b^	0.63 ± 0.08^a^	4.17 ± 0.28^c^
CSP	4.29 ± 0.08^c^	1.92 ± 0.06^b^	1.69 ± 0.07^a^
HSP	2.33 ± 0.16^c^	1.60 ± 0.14^b^	0.37 ± 0.04^a^
Dry matter^*^	94.05 ± 0.33^c^	93.02 ± 0.28^b^	88.09 ± 0.28^a^

Values are expressed as mean ± SD (*n* = 3); ^*^ expressed in g/100 g of product; SDF –soluble dietary fiber, IDF – insoluble dietary fiber, UA – uronic acid, NS – neutral sugars, KL –Klason lignins, WSP – water soluble pectins, CSP – chelator soluble pectins, HSP – hydroxide-soluble pectins; mean values within a row with different letters are significantly different at *p* < 0.05

The results obtained for the sugars analysis are shown in Table 1. Sucrose, glucose and fructose were found in fruits and flowers of VO while bark contained only sucrose. The total sugars content was the highest in VO fruits (32.27 g/100 g DW), followed by VO flowers (near three times lower) and bark with above twenty times lower content in comparison to fruit. Glucose was predominant sugar (44.8% of total sugars) in VO fruits and sucrose (47.4% of total sugars) dominated in VO flowers. Glucose was also found as dominant sugar in VO fruits by Perova et al. [5]. Previously, it has been reported that fresh VO fruits contain near 9 times more reducing sugars than sucrose [18]. However, the present results show that this ratio was about four in dried VO fruits, which may suggest quantitative changes of sugars during drying. Slatnar and co-workers [24] showed that drying methods had influence on monomer sugars/sucrose ratio in fig fruits which in fresh figs was 49.4 while in sun-drying fruits 88.2 and in oven-drying figs. 38.9.

Data in Table 1 have showed that organic acids profile and concentration depend on part of VO plant. The total acid quantified in the samples ranged from 1.81 to 7.34 g/100 g DW. Malic and citric acids were the main organic acids present in fruits, and they accounted for 84.7% of the total content. It is in agreement with data provided by Perova et al. [5] but incompatible with other studies [1, 4, 19] where malic and tartaric acids dominated. In contrast to the cited studies neither oxalic acid, fumaric nor succinic acid were detected in the fruits analyzed in our laboratory. The dominant organic acid in VO bark was succinic acid (52.7% of total content) while in flowers it was malic acid (33.7% of total content).

The content and composition of soluble (SDF) and insoluble (IDF) dietary fiber fractions varied with parts of VO plant tested (Table 1). The total dietary fiber (DF) content ranged from 38.44 g/100 g DW in fruits to 59.34 g/100 g DW in bark. The value for VO fruit is higher than data obtained for different edible fruits with DF content from 8.83 in watermelon to 38.27 g/100 g DW in cloudberry [21, 25]. The content of DF in VO flowers exceeded its level in Roselle flowers by 34% [22]. In the present study, the morphological parts of VO contained 82.3, 93.5 and 98.1% IDF of the DF in fruits, flowers and bark, respectively. The advantage of IDF over SDF content was also found in other fruits [25]. IDF is the fraction of DF which influences on consistency and stool weight and consequently reducing the intestinal transit time [26]. In all analyzed parts of VO plant, the decreasing rank of contents of particular IDF fractions was as follows: IDF KL > IDF NS > IDF UA. The SDF in fruit and bark of VO was dominated by neutral sugars (NS) fraction, while in flowers by uronic acid (UA) fraction. The SDF after ingestion is fermented by bacterial flora from gut leading to the production of the short chain fatty acids acetate, propionate and butyrate with various beneficial health effects [27]. Part of the SDF are pectins which content is shown in Table 1. Their total amounts varied significantly from 4.15 to 8.58 g/100 g DW of VO bark and flowers, respectively. Flowers and bark were dominated by ionically cross-linked pectin as reflected by the CSP (chelator-soluble pectin) value. Pectin of VO fruits mainly consisted of water-soluble pectin (WSP) which contribution in the total content of pectin in fruit and bark was the lowest.

### Natural Antioxidants of VO Fruits, Flowers and Bark

The contribution of plant-derived compounds to health improvement has been partly attributed to their antioxidant capacity. The natural plant antioxidants are mainly phenolic compounds, carotenoids, and vitamins C and E. A study was therefore undertaken to determine these phytocompounds contents in VO commercial products tested (Table 2). The statistically significant differences (*p* < 0.05) between VO dried fruits, bark and flowers in terms of total phenolics, flavonoids and proanthocyanidins were observed. Carotenoid content was the highest in VO fruits and similar in bark and flowers. The commercially available VO bark was characterized by the highest level of total phenolics, flavonoids and proanthocyanidins as compared to other parts of plant. Contrary, in none of the tested VO parts was found L-ascorbic acid. According to the literature, the concentration of ascorbic acid and carotenoids in fresh VO fruits was in the range 12.4–164.0 mg and 1.4–2.8 mg *per* 100 g fresh weight (FW) [1, 4, 5, 16, 18]. Comparing these data with our results we may conclude about the negative impact of drying on these antioxidants. Kamiloglu et al. [28] in the review of influence drying process on stability of natural antioxidants in fruits have concluded that hot-air/oven drying caused a decrease in the content of vitamin C in the range of 30–72%, and carotenoids from 0 to 90%. Degradation of vitamin C and carotenoids during drying could be attributed to their high sensitivity to oxidation, as well as the depletion of these compounds due to their utilization for protecting the oxidation of polyphenols during drying. Comparative data for dried VO fruits, bark and flowers are not available. In addition, we have not known the drying methods used to obtain the VO drought analyzed in this work.

**Table 2:** The content of antioxidants in dried flowers, bark and fruits of *Viburnum opulus*

Antioxidant	Flowers	Bark	Fruits
Carotenoids (mg β-carotene/100 g DW)	1.12 ± 0.06^a^	1.13 ± 0.03^a^	2.70 ± 0.07^b^
Phenolics total (g GAE/100 g DW)	3.51 ± 0.13^a^	3.98 ± 0.04^c^	3.73 ± 0.16^b^
Flavonoids (g CE/100 g DW)	1.67 ± 0.07^a^	2.25 ± 0.12^c^	2.01 ± 0.11^b^
Proanthocyanidins (g CYE/100 g DW)	0.22 ± 0.00^a^	1.03 ± 0.03^c^	0.52 ± 0.02^b^

Values are expressed as mean ± SD (*n* = 3). GAE – gallic acid equivalents, CE – (+)-catechin equivalents, CYE – cyanidin equivalents; mean values within a row with different letters are significantly different at *p* < 0.05

The obtained results showed that the content of total phenolics in VO different morphological parts was in the range of 3.51–3.98 g/100 g DW and significantly exceeded the concentration of carotenoids. For comparison, the content of phenolics was estimated at 0.68–0.83 g/100 g FW of VO fruits grown in Czech Republic and 0.40–0.73 g/100 g FW of fruits from Russia [5, 16]. Total phenolics in the fresh fruits grown in Turkey or in Lithuania were determined at 0.62–0.99 and 0.75–1.46 g/100 g FW, respectively [4, 18]. Total flavonoids in VO fruits found in the literature was between 0.20–0.49 g of rutin equivalents *per* 100 g FW according to colorimetric assay [4, 16], and from 0.004 to 0.255 g/100 g FW according to HPLC method [5]. In our study, total flavonoids varied from 1.67 (flowers) to 2.25 (bark) g (+)-catechin equivalents/100 g DW. Flavonoids accounted for 47.6, 53.9 and 56.5% of total phenolics in VO flowers, fruits and bark, respectively. Published data indicate that flavonoids accounted for 27.3–37.4% of the total polyphenol content in fresh VO fruits [4]. Çam et al. [19] have showed that VO seeds are better source of total phenolics and flavonoids than fruit flesh which contained 3.5- and 6.8-fold less phenolics and flavonoids. According to Perova et al. [5] proanthocyanidin content in VO fruits range from 0.20 to 0.53 g/100 g FW and they accounted for 49.9–100.0% of total phenolics. In our study, total proanthocyanidins in VO commercial products tested varied from 0.22 (flowers) to 1.03 g/100 g DW (bark) and accounted for 6.3% in flowers, 13.9% in fruits and 25.9% in bark of total phenolics.

Data on the composition of individual phenolic compounds are very important because the structure of phenolics significantly affects their properties. In connection with the above, the ethanolic extracts of VO fruits, flowers and bark were analyzed for the content of individual phenolic compounds using UPLC system. The results of qualitative and quantitative phenolic composition in VO samples are summarized in Table 3. In our study, the different biological parts of VO were characterised by a large variation in the amount of individual phenolic compounds tested. The results showed that hydroxycinnamic acids dominated in the VO fruits and flowers (88.26 and 97.23% of total phenolics) while flavanols in VO bark (80.06% of total phenolics). Additionally, we have not found flavonols in VO bark. The authors of the only report on the polyphenol composition of VO bark using one- and two-dimensional paper and thin-layer preparative chromatography have reported the presence of such acids as: caffeic, chlorogenic, *p*-hydroxybenzoic and gallic [12]. Although we have the standards of gallic, *p*-hydroxybenzoic and caffeic acids, these compounds have not been detected in dried VO bark. In the present study, a comparison of the retention time and UV-Vis absorption spectra to those of the reference compounds allowed us to determine four flavanols and four hydroxycinnamic acids in VO bark with (+)-catechin as dominated phenolic compounds (1062.43 mg/100 g DW) followed by procyanidin B1 (437.79 mg/100 g DW) and chlorogenic acid (352.49 mg/100 g DW). We provide the first report on the flavanols composition in VO bark.

**Table 3:** Content (mg/100 g DW) of phenolic compounds in dried flowers, bark and fruits of *Viburnum opulus*

Compound	Flowers	Bark	Fruits
Procyanidin B1	25.46 ± 0.10^b^	437.79 ± 0.19^c^	14.02 ± 0.64^a^
(+)-Catechin	46.87 ± 0.11^a^	1062.43 ± 1.27^b^	–
Procyanidin B2	–	116.47 ± 0.48	–
(−)-Epicatechin	–	95.87 ± 1.00	–
Total flavanols	72.33 ± 0.18^c^	1712.55 ± 2.23^1712^	14.02 ± 0.64^b^
Neochlorogenic acid	17.22 ± 0.03^b^	41.51 ± 0.04^c^	7.22 ± 0.22^a^
Chlorogenic acid	1535.42 ± 5.53^c^	352.49 ± 0.33^a^	752.59 ± 2.07^b^
Cryptochlorogenic acid	6.78 ± 0.01b	18.44 ± 0.30^c^	3.51 ± 0.01^a^
*p*-Coumaric acid	–	14.17 ± 0.25	–
Total hydroxycinnamic acid	1559.42 ± 5.56^1559^	426.61 ± 0.33^a^	763.32 ± 2.07^b^
Rutin	10.05 ± 0.02^b^	–	5.39 ± 0.03^a^
Isorhamnetin	58.84 ± 0.10^b^	–	0.71 ± 0.01^a^
Isorhamnetin 3-O-rutinoside	–	–	1.60 ± 0.02
Isorhamnetin 3-O-glucoside	47.06 ± 0.08	–	–
Quercetin 3-O-glucoside	19.18 ± 0.04	–	–
Total flavonols	135.13 ± 0.23^b^	–	7.70 ± 0.02^a^
Sum of phenolic compounds	1766.88 ± 5.97^1776^	2139.16 ± 2.36^2139^	784.94 ± 1.52^a^

Values are expressed as mean ± SD (*n* = 3), mean values within a row with different letters are significantly different at *p* < 0.05

Regarding the most studied phenolic profile of VO fruit, hydroxybenzoic and hydroxycinnamic acids, catechins and procyanidins as well as flavonols and anthocyanins were identified by HPLC-MS-TOF and HPLC methods [1, 3, 5]. However, the results of quantitative analysis are controversial. According to Özrenk et al. [1] the most abundant components in fruits were (+)-catechin (28–35 mg/100 g FW) and gallic acid (11–12 mg/100 g FW) while chlorogenic acid concentration was 8–10 times lower than the (+)-catechin content. On the other hand, Velioglu et al. [3] indicated chlorogenic acid (204 mg/100 g FW) as the main component of VO fruits followed by (+)-catechin (29 mg/100 g FW). In the present study, chlorogenic acid (752.59 mg/100 g DW) dominated in VO dried fruits tested. Additionally, we have found in VO fruits quercetin and isorhamnetin glycosides like other authors [3, 5]. Unfortunately, in the present study, the anthocyanins have not been found in dried VO fruits although they were identified in fresh fruits [4, 5, 18]. Published data indicate that VO fruits contain the different cyanidin glycosides which level ranged between 10 and 31 mg/100 g FW [5]. It can be assumed that the lack of anthocyanins in dried VO fruits may have resulted from the drying process of fruit. The hot-air/oven drying of different fruits decreased the total anthocyanin content in the range of 42–92% [28]. For example, air-drying at 62–64 °C for 24 h caused 26–61% decreases in cyanidin-3-rutinoside content in fig, while drying at 70 °C resulted in 97% loss of cyanidin-3-glucoside in red guava, whereas sour cherry dried at 50–70 °C contained 27–38% less cyanidin-3-glucoside compared to the fresh fruits.

There are no previous studies and references on the phenolics composition of the VO flowers. We report here the first UPLC profile of VO flowers. According to our data, flowers were more abundant in hydroxycinnamic acids, flavonols, and flavanols compared to fruits. The main phenolic compounds in flowers was chlorogenic acid (1535.42 mg/100 g DW) followed by isorhamnetin (58.84 mg/100 g DW), isorhamnetin 3-glucoside (47.06 mg/100 g DW), and (+)-catechin (46.87 mg/100 g DW).

### Antioxidant Capacity of VO Fruits, Flowers and Bark

The use of plants rich in antioxidants can prevent or delay the development of non-communicable diseases through processes involving reactive oxygen species. In biological system, reactive oxygen species, such as superoxide, hydroxyl radicals, can damage the DNA and lead to the oxidation of cellular lipid and proteins [29]. Antioxidant properties of VO flowers, bark and fruits were estimated by five different methods, as scavenging potential toward stable, synthetic ABTS^•+^ radical cation (ABTS) and toward reactive oxygen species such as ^•^OH radical (HORS), O_2_^•-^ superoxide anion radical (SORS), peroxyl radical (ORAC) and as the potential to reduce ferric to ferrous ion (FRAP). Trolox – a water-soluble analog of vitamin E, was used as an antioxidant standard to determine the Trolox equivalent (TE). Exceptionally, (+)-catechin was used as the antioxidant standard in the SORS assay to determine the catechin equivalent (CE). The higher TE and CE values, the greater the antioxidant potential. The results for antioxidant capacity of VO natural antioxidants, extractable by 70% ethanol, are presented in Table 4. Significant differences (*p* < 0.05) were found among the analyzed parts of VO in the antioxidant capacity, except VO flowers and fruits in SORS assay. The TE values varied from 16.18 to 40.21 mM/100 g DW of product in ABTS assay, from 5.91 to 10.05 mM/100 g DW in HORS method, from 10.93 to 108.17 mM/100 g DW in ORAC assay, and in the range 13.65–23.47 mM/100 g DW in FRAP method. Antioxidant potential of VO products in SORS assay as CE ranged between 89.77 and 115.44 mM/100 g DW of product. The antioxidant capacity of VO different parts investigated was in the following order in HORS, SORS and ORAC assays: bark > flowers > fruits and the order in ABTS and FRAP assays was bark > fruits > flowers. The values obtained clearly reflect that the VO bark components showed the greatest antioxidant capacity, regardless of the method used. The scavenging effect of VO fruit components on ABTS^•+^, DPPH^•^ (2,2-diphenyl-1-picrylhydrazyl), ^•^OH, O_2_^•-^ and NO^•^ (nitric oxide) radicals are reported in the literature [11, 16, 19]. Rop et al. [16] noticed the cultivar variability in case of scavenging effect of VO fruits on different free radicals. The antioxidant capacity of VO fruits toward O_2_^•-^ radical was lower than VO branch and leaf activities but higher than VO leaf activity in DPPH assay [11]. In contrast to fruits, the antioxidant capacity of VO bark was determined only by Andreeva et al. [12] using the method of cathode voltammetry. According to our knowledge, there is no data on the antioxidant properties of VO flowers. The TE values for VO flowers observed in the present study are about twice and three times higher than those obtained for Roselle flowers in ABTS, FRAP and ORAC assays, respectively [22]. Correlations between antioxidant activity assays with phenolic compounds tested by colorimetric assays (Table 2) are presented in Table 5. High correlations were found between total phenolics, flavonoids and proanthocyanidins with ABTS and FRAP assays (*r* ≥ 0.97). Correlation analysis also evidenced a positive relationship between the total phenolic, flavonoid and proanthocyanidin contents with SORS test (*r* ≥ 0.78). On the other hand, the Pearson’s coefficients listed in Table 5 suggest week correlation between the phenolic compound contents and ORAC assay and negative correlation with HORS test.

**Table 4:** Antioxidant capacity of dried flowers, bark and fruits of *Viburnum opulus*

Assay	Unit	Flowers	Bark	Fruits
ABTS	mM TE/100 g DW	16.18 ± 1.35^a^	40.21 ± 0.67^c^	26.57 ± 1.71^b^
HORS	mM TE/100 g DW	8.23 ± 0.39^b^	5.91 ± 0.33^a^	10.05 ± 0.31^c^
ORAC	mM TE/100 g DW	61.82 ± 2.04^b^	108.17 ± 3.38^c^	10.93 ± 0.39^a^
FRAP	mM TE/100 g DW	13.65 ± 0.64^a^	23.47 ± 1.50^c^	19.29 ± 0.83^b^
SORS	mM CE/100 g DW	91.13 ± 3.40^a^	115.44 ± 5.28^b^	89.77 ± 2.56^a^

Values are expressed as mean ± SD (*n* = 3), TE – trolox equivalents, CE – (+)-catechin equivalents, mean values within a row with different letters are significantly different at *p* < 0.05

**Table 5:** Pearson’s correlation coefficients (*r*) between total phenolics, flavonoids and procyanidins content and antioxidant capacity of *Viburnum opulus* commercial products

	ABTS	FRAP	ORAC	HORS	SORS
Total phenolics	0.999	0.993	0.510	−0.589	0.861
Flavonoids	0.984	1	0.389	−0.474	0.784
Proanthocyanidins	0.998	0.973	0.603	−0.676	0.912

## Conclusion

In conclusion, this research has comprehensively investigated the chemical composition and antioxidant capacity of different parts (fruits, flowers and bark) of *Viburnum opulus* (VO) which are commercially available in Poland. This is the first detailed report on the phytochemicals and antioxidant properties of VO flowers as well as on the macronutrients in VO bark. The results demonstrated the differences in the basic chemical and bioactive compounds composition, and antioxidant properties of different morphological parts of VO. Bark constituents were the most active against free radicals, which was consistent with its highest phenolic compounds contents, especially proanthocyanidins and flavanol monomers. In addition, our results indicate that VO bark and flowers have high commercial potential due to their higher dietary fibre and phenolics contents, and lower sugars concentration in comparison to VO fruits. Taking into account numerous published data on the relationship between the biological activity, especially antioxidant properties, and the content of phenolic compounds for future studies, it is necessary to investigate the phenolic compound profile of VO bark and flowers using mass spectrometry method.

## Electronic supplementary material

ESM 1 (DOCX 26 kb)

## Abbreviations

VO: *Viburnum opulus*
ABTS: ABTS^•+^ radical cation scavenging capacity
CE: (+)-catechin equivalents
CSP: Chelator soluble pectins
CYE: Cyanidin equivalents
DW: Dry weight
FRAP: Ferric reducing power
FW: Fresh weight
GAE: Gallic acid equivalents
HORS: Hydroxyl radical scavenging capacity
HSP: Hydroxide-soluble pectins
IDF: Insoluble dietary fiber
KL: Klason lignins
NS: Neutral sugars
ORAC: Oxygen radical scavenging capacity
SDF: Soluble dietary fiber
SORS: Superoxide anion radical scavenging capacity
TE: Trolox equivalents
UA: Uronic acid
WSP: Water soluble pectins
