# Supplementary material for: Comparison of Chemical Composition and Antioxidant Capacity of Fruit, Flower and Bark of Viburnum opulus
Source: Plant Foods Hum Nutr. 2019 Jul 19;74(3):436–42. doi: 10.1007/s11130-019-00759-1 (PMC6684545; doi:10.1007/s11130-019-00759-1)
Supplement: Supplementary file 1 — (DOCX 26 kb) [file 11130_2019_759_MOESM1_ESM.docx]

SUPPLEMENTARY MATERIAL

**Article title:** Comparison of Chemical Composition and Antioxidant Capacity of Fruit, Flower and Bark of *Viburnum opulus*

**Journal name**: Plant Foods for Human Nutrition

**Author names**: Dominika Polka, Anna Podsędek^*^, Maria Koziołkiewicz

#### Affiliation and e-mail address of the corresponding author: Institute of Technical Biochemistry, Department of Biotechnology and Food Sciences, Lodz University of Technology, Stefanowskiego 4/10, 90-924 Łódź, Poland; e-mail: [anna.podsedek@p.lodz.pl](mailto:anna.podsedek@p.lodz.pl)

**Materials and Methods**

**Standards and Reagents**

2,2’- Azobis(2-amidinopropane) dihydrochloride (AAPH), 2,2’-azinobis(3-ethyl-benzthiazoline-6-sulphonic acid) (ABTS), D(+)-galacturonic acid, 2-deoxy-D-ribose, d**iphosphopyridine nucleotide-reduced form (NADH), 3,5-dimethylphenol,** ethylenedi-aminetetraacetic acid **(EDTA),** fluorescein, 6-hydroxy-2,5,7,8-tetramethychroman-2-carboxylic acid (Trolox), iron(III) chloride (FeCl_3_), meta-phosphoric acid, methanol, nitroblue tetrazolium (NBT), potassium persulfate (K_2_S_2_O_8_), thiobarbituric acid (TBA), trichloroacetic acid (TCA), 2,4,6-tris-2-pyridyl-*s*-triazine (TPTZ), and **phenazine methosulfate (PMS)** were obtained from Sigma-Aldrich (Steinheim, Germany). Acetonitrile (Merck, Germany) and formic acid (Sigma-Aldrich) were hyper grade for LC-MS. Aluminium chloride (AlCl_3_), buthanol, hexane, hydrogen chloride (HCl), hydrogen peroxide (H_2_O_2_), sodium carbonate (Na_2_CO_3_), sodium nitrite (NaNO_2_), sodium hydroxide (NaOH) were purchased from Chempur (Piekary Śląskie, Poland). Folin-Ciocalteu reagent, hexane and ethanol were obtained from POCH (Gliwice, Poland). Reference compounds were obtained from Sigma-Aldrich (Steinheim, Germany) (ascorbic acid, β-carotene, (+)-catechin, caffeic acid, (-)-epicatechin, rutin, fructose, gallic acid, glucose, sucrose, citric acid, fumaric acid, malic acid, oxalic acid, quinic acid, succinic acid and tartaric acid), Extrasynthese (Lyon, France) (chlorogenic acid, *p*-coumaric acid, cyanidin 3-glucoside, quercetin 3-glucoside, isorhamnetin, isorhamnetin 3-glucoside, and isorhamnetin 3-rutinoside), and Phytolab (Vestenbergsgreuth, Germany) (neochlorogenic acid, cryptochlorogenic acid, procyanidin B1, and procyanidin B2). Ultrapure water (Simplicity^TM^ Water Purification System, Millipore, Marlborough, MA, USA) was used to prepare all solutions.

**Plant material**

The commercial products of *V. opulus*, such as dried flowers, fruits and bark were purchased from “Nanga Przemysław Figura” herbal wholesaler (Złotów, Poland), “Natura Wita Ltd” (Kopernia, Poland) and “Farm Vit” (Szczecin, Poland), respectively. Droughts were grind in a coffee grinder and powders were kept in tightly closed containers in a laboratory cabinet without light.

##### **Analysis of elementary chemical composition**

##### Dry matter, ash, protein and fat contents were determined by standard procedures [1]. Moisture was determined by drying in an oven with air circulation at 105 °C to constant weight and was expressed as gram per 100 g of VO commercial product. Ash was determined as the weight loss after incineration of samples in a muffle furnace at 600 °C for 6 h. Crude protein content was analyzed by the Kjeldahl method with a conversion factor of 6.25. Crude fat as hexane extract was determined using a Soxhlet extractor. The flask containing hexane extract was dried at 40 ºC under vacuum, and the weight of the crude fat was noted. The results were expressed as gram of analyzed compound per 100 g dried weight (DW) of VO commercial product.

**Extraction and analysis of sugars and organic acids**

Ground plant materials (0.2 g) with 25 mL of water were boiled and then cooled. The volume of each sample was adjusted to 25 mL and filtered through a paper filter. Ten millilitres of filtrate was mixed with 2.5 mL of Carrez I and II solutions. After shaking the solution was kept at the room temperature for 10 min and adjusted to 25 mL with water. The clarified sample mixture was centrifuged at 5000 rpm for 10 min. Individual sugars and organic acids in supernatants were determined by the HPLC system with a photodiode array detector and a refractive index detector connected in series (Waters Corp., Milford, MA, USA). Separation was achieved on an ion exclusion Rezex ROA-Organic H^+^ column (300 x 7.8 mm, Phenomenex) according to the procedure described by Aubert et al. [2]. The elution system was 0.005 N H_2_SO_4_, running isocratically at a flow rate 0.4 mL/min. Organic acids were quantified from the absorbance peaks at 210 nm and using calibration curves performed with citric, fumaric, malic, oxalic, quinic, succinic and tartaric acid standards. Sugars were quantified by comparison of refractive index peaks with those of standards of fructose, glucose and sucrose. The results were expressed as gram of individual sugar or organic acid per 100 g DW of VO commercial product.

**Determination of dietary fiber (DF) profiling**

Total DF was determined as the sum of soluble (SDF) and insoluble (IDF) dietary fiber in the VO commercial products deprived of fat, mono- and oligosaccharides, and protein, according to Gouw et al. [3]. The content of SDF was the sum of uronic acids (SDF UA) and neutral sugars (SDF NS) determined by spectrophotometric method in the filtrate after acid hydrolysis of VO samples. The IDF concentration was evaluated as the sum of IDF UA and IDF NS in the residue after acid hydrolysis of VO samples and the mass of Klason lignins (KL). KL was quantified gravimetrically reduced by the ash mass.

**Pectin analysis**

Total pectin was determined as the sum of water soluble (WSP), chelator soluble (CSP) and hydroxide-soluble (HSP) pectin according to Gouw et al. [3]. WSP was evaluated gravimetrically after precipitation with ethanol, CSP and HSC were determined by spectrophotometric method as uronic acid (UA) .

**Extraction and analysis of total carotenoids**

The VO products (0.2 g) were extracted using hexane until the organic layer was colourless. Mixed organic layers were measured at 450 nm against the solvent blank, and total carotenoids content was expressed as milligrams of β-carotene equivalents per 100 g DW of VO products.

**Extraction and analysis of ascorbic acid**

The VO products (0.1 g) were extracted with a 1% solution of meta-phosphoric acid (10 mL) in an ultrasonic bath for 30 min at room temperature and centrifuged at 5500 rpm for 10 min. Then, the samples were analyzed using a high-performance liquid chromatography system (Waters, Milford, MA) that consisted of a gradient pump (1525), photodiode array detector (2998), auto-injector (2707) and Breeze 2 system controller equipped with a 250 x 4.6 mm i.d, 5 µm Symmetry C18 column (Waters). The HPLC method was adapted from Gliszczynska-Swiglo and Tyrakowska [4]. For analysis of ascorbic acid the following gradient of methanol (solvent A) and 5 mM KH_2_PO_4_ pH 2.6 (solvent B) was used: linear increase of solvent A from 5 to 22% in 6 min and then return to the initial conditions within the next 9 min with a flow rate of 1 mL/min with a UV‑Vis detector set at 245 nm. Ascorbic acid was identified by comparison with its genuine sample.

**Sample extraction for phenolic compounds and antioxidant activity assays**

Grounded commercial products of *V. opulus* were extracted with 70% ethanol (1:20, w/v) on a magnetic stirrer at room temperature for 3 h. Then, the mixtures were incubated at room temperature for 18 h followed by the extraction on a magnetic stirrer at room temperature for 3 h. After centrifugation at 5000 rpm for 10 min the supernatants were evaporated at 40 ºC under reduced pressure in order to remove ethanol. The aqueous phases were lyophilized to dryness to obtain the crude extracts which were further analyzed for phenolic compounds content and antioxidant capacity. Stock solution of each crude extract was prepared at concentration 25 mg/mL in water.

**Quantification of phenolic compounds by spectrophotometric methods**

Total phenolics content was determined using Folin-Ciocalteu reagent as we described in our previous work and was expressed as mg of gallic acid equivalents (GAE) per 100 g DW of VO product [5]. Total flavonoid content was determined spectrophotometrically using a method based on the formation of a complex flavonoid-aluminium [6]. The results were expressed as mg of (+)-catechin equivalents (CE) per 100 g DW of VO product. The content of total proanthocyanidins was determined after their acid depolymerization to the corresponding anthocyanidins as described by Rösch et al. [7] and calculated by the molar extinction coefficient of cyanidin (ε = 17360 l/mol* cm and molar mass 287 g/mol), and was expressed as gram of cyanidin equivalents (CYE)/100 g DW of VO product.

**Individual phenolic compounds determination**

Phenolic profiles were determined using an ACQUITY Ultra Performance LC system (UPLC) equipped with a photodiode array detector with a binary solvent manager (Waters, Milford, MA). The data were collected by Mass-LynxTM V 4.1 software. Separation was achieved on a Acquity UPLC HSS T3 column (150 x 2.1 mm, 1.8 µm; Waters). The mobile phase was a binary gradient with A, water/formic acid (95.5:4.5, v/v), and B, acetonitrile, with a flow rate of 0.45 mL/min [8]. The binary gradient was as follows: initial conditions - 99% A (0 min), 12 min - 75% A, 12.5 min -100% B, 13.5 min - 99% A (12.5-13.5 min). The runs were monitored at the following wavelengths: flavanols at 280 nm, hydroxycinnamic acids at 320 nm, flavonols at 360 nm and anthocyanins at 520 nm. The retention times and spectra were compared to those of the authentic standards. Calibration curves at concentrations ranging from 0.06 to 2 mg/mL (r^2^ ≥ 0.96) were made from caffeic acid, chlorogenic acid, gallic acid, neochlorogenic acid, cryptochlorogenic acid, *p*-coumaric acid, (+)catechin, (-)epicatechin, procyanidins B1 and B2, cyanidin 3-glucoside, quercetin 3-glucoside, rutin, isorhamnetin, isorhamnetin 3-glucoside, isorhamnetin 3-rutinoside. The results were expressed as mg/100 g DW of VO product.

**In vitro antioxidant activity assays**

Antioxidant capacity of VO commercial products was determined by ABTS radical cation (ABTS), hydroxyl radical (HORS), superoxide anion radical (SORS) and oxygen radical (ORAC) scavenging capacity, and as ferric reducing power (FRAP) using ethanolic extracts of products tested (section 2.9). Antioxidant activity was expressed as mM Trolox equivalents (TE) per 100 g DW of VO product except superoxide anion radical scavenging activity which was expressed as mM (+)-catechin equivalents (CE) per 100 g DW of VO product.

ABTS and FRAP assays procedures have been detailed in our previous work [5]. HORS assay was provided according to Racchi et al. [9] with some modifications. The reaction mixture contained 2.5 mL of phosphate buffer (20 mM, pH 7.4), 0.1 mL of 18 mM 2-deoxy-D-ribose, 0.1 mL of 6 mM FeCl_3_, 0.1 mL of 6 mM EDTA, 0.05 mL of 12 mM H_2_O_2_, 0.1 mL of 0.6 mM ascorbic acid, and 0.05 mL of ethanol extract, or the same volume of buffer (control sample). For each sample, a separate blank without 2-deoxy-D-ribose was used for background subtraction. Samples were incubated at 37 °C for 1 h, and then 1 mL of 1% TBA and 1 mL of 2.8% TCA were added. The reaction mixtures were heated at 80 °C for 30 min, kept in ice for 5 min, and then measured at 532 nm against a solution prepared as described but without 2-deoxy-D-ribose and ethanol extract.

In SORS assay the effect of the control and ethanol extracts of VO products on the superoxide radical anion (O_2_^●-^) induced reduction of NBT at 560 nm. O_2_^●-^ were generated by the NADH/PMS system according to procedure described by Sreerama et al. [10].

ORAC assay was carried out according to Kevers et al. [11] by using a microplate reader (Synergy^TM^2, BioTek Instruments Inc.). Peroxyl radicals were generated by the spontaneous decomposition of AAPH at 37 ºC. The loss of fluorescence of fluorescein was an indication of the extent of damage from its reaction with the peroxyl radicals. Fluorescence was read at 485 nm excitation and 520 nm emission every 2 min for 2 h.

**Statistical analysis*.*** Data from the present study was presented as the mean ± standard deviations of least three replicates for each sample. Differences between groups were tested by one-way analysis of variance (ANOVA) followed by Duncan’s post hoc test. Statistically significant diferences were set at *p* < 0.05.

**References**

1. Nollet LM (2004) Physical Characterization and Nutrient Analysis. In: Handbook of Food Analysis, 2nd edn. Marcel Dekker, New York, pp 1, 61-67, 77-78, 173-176, 233.
2. Aubert C, Bony P, Chalot G, Landry P, Lurol S (2014) Effects of storage temperature, storage duration, and subsequent ripening on the physicochemical characteristics, volatile compounds, and phytochemicals of western red nectarine (*Prunus persica* L. Batsch), J Agric Food Chem 62: 4707-4724.
3. Gouw VP, Jung J, Zhao Y (2017) Functional properties, bioactive compounds, and in vitro gastrointestinal digestion study of dried fruit pomace powders as functional food ingredients, LWT-Food Sci Technol 80: 136-144.
4. Gliszczynska-Swiglo A, Tyrakowska B (2003) Quality of commercial apple juices evaluated on the basis of the polyphenol content and the TEAC antioxidant activity, J Food Sci 68: 1844-1849.
5. Podsędek A, Majewska I, Redzynia M, Sosnowska D, Koziołkiewicz M (2014) In vitro inhibitory effect on digestive enzymes and antioxidant potential of commonly consumed fruits. J Agric Food Chem 62: 4610-4617.
6. Sakanaka S, Tachibana Y, Okada Y (2005) Preparation and antioxidant properties of extracts of Japanese persimmon leaf tea (kakinoha-cha). Food Chem 89: 569-575.
7. Rӧsch D, Bergmann M, Knorr D, Kroh LW (2003) Structure – antioxidant efficiency relationships of phenolic compounds and their contribution to the antioxidant activity of sea buckthorn juice. J Agric Food Chem 51: 4233-4239.
8. Wojnicz D, Kucharska AZ, Sokół-Łętowska A, Kicia M, Tichaczek-Goska D (2012) Medicinal plants extracts affect virulence factors expression and biofilm formation by the uropathogenic *Escherichia coli*. Urol Res 40: 683-697.
9. Racchi M, Daglia M, Lanni C, Papetti A, Govoni S, Gazzani G (2002) Antiradical activity of water soluble components in common diet vegetables. J Agric Food Chem 50: 1272-1277.
10. Sreerama YN, Takahashi Y, Yamaki K (2012) Phenolic antioxidants in some *Vigna* species of legumes and their distinct inhibitory effects on α-glucosidase and pancreatic lipase activities. J Food Sci 77: 927-933.
11. Kevers C, Falkowski M, Tabart J, Defraigne J-O, Dommes J, Pincemail J (2007) Evaluation of antioxidant capacity during storage of selected fruits and vegatables. J Agric Food Chem 55: 8596-8603.
